# Robust assignment of cancer subtypes from expression data using a uni-variate gene expression average as classifier

**DOI:** 10.1186/1471-2407-10-532

**Published:** 2010-10-06

**Authors:** Martin Lauss, Attila Frigyesi, Tobias Ryden, Mattias Höglund

**Affiliations:** 1 Department of Oncology, Clinical Sciences, Lund University and Lund University Hospital, SE-221 85 LUND, Sweden; 2 Department of Anesthesiology and Intensive Care, Lund University Hospital, SE-221 85 Lund, Sweden; 3 Centre for Mathematical Sciences, Lund University, Box 118, SE-221 00 Lund, Sweden

## Abstract

**Background:**

Genome wide gene expression data is a rich source for the identification of gene signatures suitable for clinical purposes and a number of statistical algorithms have been described for both identification and evaluation of such signatures. Some employed algorithms are fairly complex and hence sensitive to over-fitting whereas others are more simple and straight forward. Here we present a new type of simple algorithm based on ROC analysis and the use of metagenes that we believe will be a good complement to existing algorithms.

**Results:**

The basis for the proposed approach is the use of metagenes, instead of collections of individual genes, and a feature selection using AUC values obtained by ROC analysis. Each gene in a data set is assigned an AUC value relative to the tumor class under investigation and the genes are ranked according to these values. Metagenes are then formed by calculating the mean expression level for an increasing number of ranked genes, and the metagene expression value that optimally discriminates tumor classes in the training set is used for classification of new samples. The performance of the metagene is then evaluated using LOOCV and balanced accuracies.

**Conclusions:**

We show that the simple uni-variate gene expression average algorithm performs as well as several alternative algorithms such as discriminant analysis and the more complex approaches such as SVM and neural networks. The R package *rocc *is freely available at http://cran.r-project.org/web/packages/rocc/index.html.

## Background

One of the most promising clinical applications of genome wide expression studies is the construction of robust and reliable disease classifiers. Correct identification and sub-classification of diseases such as cancer is a prerequisite for proper and efficient treatment. To date a large number of different algorithms for disease classification have been described. They range in complexity from neural network approaches [1] to the simpler nearest-neighbor classification algorithms [2]. Even though some of the more complex approaches such as neural networks and self organized maps (SOM) [3] have proved to be very efficient, these methods often rely on the tuning of several parameters and hence are liable for over-fitting. Furthermore, simple classifiers seem to perform remarkably well when compared to more sophisticated ones [4]. In the present investigation our aim has been to design a simple predictor system useful for cancer subtype classification. Features to be included in the predictor signatures are selected based on their classification capacity as determined by a receiver operating characteristic (ROC) analysis and area under the curve (AUC) estimates [5,6]. After selection of the appropriate number of genes in the predictor signature, the mean expression level of all genes included is calculated, transforming the ensemble of genes into one vector and used as a uni-variate gene expression average, or a metagene, as classifier. Two features of gene expression are exploited by the merging of genes, genes are often co-regulated and hence correlated, and by using the *expression level *of the metagene, effects by random noise from single genes are minimized. Most of the commonly used algorithms such as SVM [7] and PAM [8] apply specifications such as support vectors and weights to the individual features included in the predictor gene signatures which potentially complicate their application to independent data [9]. Hence in this investigation we use an alternative way to evaluate the results by using the obtained training set gene signature genes only and then establish new parameters in the validation set to evaluate the performance of the classifier. We show that the proposed metagene classifier produces excellent accuracies, similar to what is obtained with a SVM approach, in several types of cancer data sets using a variety of tumor classification criteria.

## Implementation

### Data sets

To establish the classifier we used bladder cancer datasets produced by Sanchez-Carbayo et al. [10] (Supplementary Table 10 in [10]) "SanchezC", Stransky et al. [11] (ArrayExpress: E-TABM-147) "Stransky"; and Blaveri et al. [12] (Supplementary Table 4 in [12]) "Blaveri". The remaining datasets were obtained from Gene Expression Omnibus (GEO) [13], except for the vandeVijver breast cancer dataset [14]. The following datasets were downloaded from GEO; for breast GSE2034 (WangY), GSE2990 (Sotiriou), for neuroblastoma GSE3960 (WangQ), GSE12460 (JanoueixL), GSE19274 (Attiyeh), for lung GSE8569 (Angulo), GSE11969 (Takeuchi). For a detailed description of the datasets see Additional file 1. Normal urothelium samples, recurring tumors from the same patient, cell lines, and technical replicates were not included in the final bladder cancer data sets. The SanchezC dataset was quantile-normalized using the normalizeBetweenArrays function of the R package *limma *[15]. Robust Multi-array Average (RMA) was performed separately for two samples sets of the Stransky dataset (on U95A and U95Av2 respectively) using the *affy *package [16]. Obtained RMA expression values were de-logged, the samples sets combined, and quantile normalized using *limma*. The SanchezC and Stransky datasets were both transformed to log2 scale. To obtain gene-centered values the gene expression values were subtracted by the mean expression of the gene in each dataset separately. The Blaveri dataset was imputed for missing values using k-nearest neighbors (k = 10) for genes that had no more than 20% missing data, and genes with >20% missing data were omitted [17]. The HGNC GeneSymbols were updated in all datasets with the official HGNC GeneSymbols from the HGNC webpage [18]. The expression values of GeneSymbols with multiple reporters were merged by taking the median expression value. All reporters in the datasets without a GeneSymbol were discarded. The final SanchezC dataset contained 90 patients and 12761 genes, the Stransky dataset 56 patients and 8955 genes, and the Blaveri dataset 74 patients and 4430 genes. The SanchezC and Stransky datasets share a total of 8518 GeneSymbols and were used to explore the AUC characteristics. For classification, Ta and T1 cases were considered non-muscle invasive (NMI), and ≥T2 cases as muscle-invasive (MI). Grade is discriminated between Grade 2 and 3 in SanchezC, and between Grade1+2 and Grade 3 in Sanchez. Randomized versions of the datasets were generated using the mean and standard deviation of the original datasets. Non-bladder cancer Affymetrix datasets not already normalized were downloaded as cel files and normalized using RMA. All other datasets were downloaded as normalized 'series matrix files'. In the case of missing values, k-nearest neighbor imputation was performed (k = 10). Gene Symbols were updated using the official HGNC nomenclature file, and then expression values for reporters with the same GeneSymbols were merged. The data was mean-centered, except for two-color array data, as this data comes already in ratios. Reporters with no GeneSymbols were excluded from the final data. The vandeVijver data was imputed for missing values by k-nearest neighbor, transformed to log2 ratios and GeneSymbols were updated and merged.

### ROC analyses

The receiver operating characteristic (ROC) curve is the plot of sensitivity (true positive rate) vs. 1-specificity (false positive rate), for predicting a binary classification variable *z *using some covariate *x*. That is, if *x *>*t *for some threshold *t *then *z *is predicted as 1, otherwise as 0. As the threshold t ranges from *+*ℕ to -ℕ the fraction of true positive and false positive predictions will both increase from 0 to 1, yielding the ROC curve. The area under this curve (AUC) is an overall measure of the predictor's performance. An ideal predictor obtains true positive rate 1 and false positive rate 0 for some threshold *t*, and then AUC = 1. Ignoring covariate information and guessing randomly by predicting *z *= 1 with some probability *q *yields AUC = 1/2 by letting *q *range from 0 to 1, so 1/2 is a worst-case AUC. If AUC < 1/2 the covariate *x *is however negatively correlated with *z*, and replacing *x *by -*x *turns AUC into 1-AUC (which will be >1/2). For a given covariate *x *we can thus view max(AUC,1-AUC) as its performance. For a finite sample of x's and z's, AUC can be computed as a function of the Mann-Whitney (or two-sample Wilcoxon) statistic for comparing the *x*'s associated *z *= 0 and *z *= 1 respectively.

### Supervised classification using the uni-variate gene expression average classifier

Genes to be included in the gene signatures are selected in order of their ranked max(AUC,1-AUC) values. To merge the gene expression of a given gene signature to a single metagene expression value, an arithmetic mean is computed by summing up the expression values after multiplying expression values for genes negatively associated with the feature (AUC < 0.5) by -1 (Additional file 2). The resulting metagene expression values are then used in ROC analyses, i.e. by ranking the samples according to their metagene expression values. The optimal split of positive (i.e., 1) and negative (i.e., 0) samples is determined as the metagene expression threshold which produces the highest accuracy i.e., correct class assignments in respect to the real class, in the training set. More precisely, the threshold is computed as the mean metagene expression value of the two samples that constitute the border of the split. A new sample to be classified has its metagene expression value determined with the same genes to be multiplied by -1. The new sample is classified according to which side of the threshold the sample falls in, with a sample having higher metagene expression being classified as positive (i.e., 1) and with lower expression as negative (i.e., 0). The split yielding optimal accuracy in the ROC curve is determined using the R package *ROCR *[19]. The outlined approach is to some respect similar to the approach described by Rosenwald et al [20] except that we have simplified the use of metagenes further by using one single metagene and thus do not have to assign any specific weights to individual metagenes. In addition we optimize the threshold for each dataset.

### Additional classification algorithms

We compared our classifier initially to a Support Vector Machine (SVM) with a linear kernel. SVMs have been shown to perform considerably well in microarray data [21] and SVMs with a linear kernel has been suggested to perform better in gene expression data than more complex SVM versions (Manual BRB Array Tools, [22]), and additionally, no parameter tuning is necessary for linear SVMs. Briefly, Support Vector Machines (SVMs) identify the maximum margin hyperplane that optimally separates the training samples (based on support vectors) and then classify unseen samples according to the side of the hyperplane they fall into [23]. We used the 'svm' function of the R package *e1071 *[24]. Additional classification algorithms were implemented using the R package *MLInterfaces *[25] using the default settings and included SVM with radial kernel (SVMradial), SVM with polynomial kernel (SVMpoly), k-nearest neighbor (knn, k = 3), random forest (rforest), recursive partitioning trees (rpart), bagging (bagging), linear discriminant analysis (lda), diagonal linear discriminant analysis (dlda), stabilized linear discriminant analysis (slda), neural network (neural net, with 3 hidden layers), except for the nearest centroid classifier (ncc) that was implemented using the *pamr *package [8].

### Performance Evaluation

As the accuracies of prediction are dependent on the prior distribution of samples, we used balanced accuracies computed by *(sensitivity + specificity)/2*. Balanced accuracies are independent of the prior distributions [26]. Unbiased accuracies were obtained by leave-one-out-cross-validation (LOOCV). Feature selection was repeated in each loop of LOOCV. When testing the performance of a gene signature in independent validation data only the genes were used in the validation data, i.e. not the information on AUC and 1-AUC or the classification threshold. In a clinical validation platform, as qPCR and IHC, we assume that the classifier specification might not be taken over. We applied a LOOCV loop to determine max(AUC,1-AUC) and optimal classification threshold for the signature metagene in each loop. Accuracies obtained from cross-validation loops are an estimate for the accuracy obtained from the whole dataset, i.e. all samples.

### R package rocc

Briefly, the package includes the functions tr.rocc, p.rocc, and o.rocc. The function tr.rocc uses a training set with a given phenotype to determine the metagene threshold. The function p.rocc predicts the class of a new sample using the classifier specification of the tr.rocc output. The function o.rocc performs a LOOCV loop using a metagene of given size, e.g., top 200 genes, with feature selection in each loop separately.

tr.rocc (data,out,xgenes = 200)

p.rocc (tr.rocc.object,newsample)

o.rocc (data,out,xgenes = 200)

*data *= dataset as a matrix file with samples as columns and genes as rows

*out *= phenotype as factor with levels 0 and 1

*xgenes *= number of genes that constitute the metagene; can be a numeric vector.

*newsample *= sample to be classified using a classifier obtained from tr.rocc()

## Results and Discussion

### Feature selection

**Figure 1:**
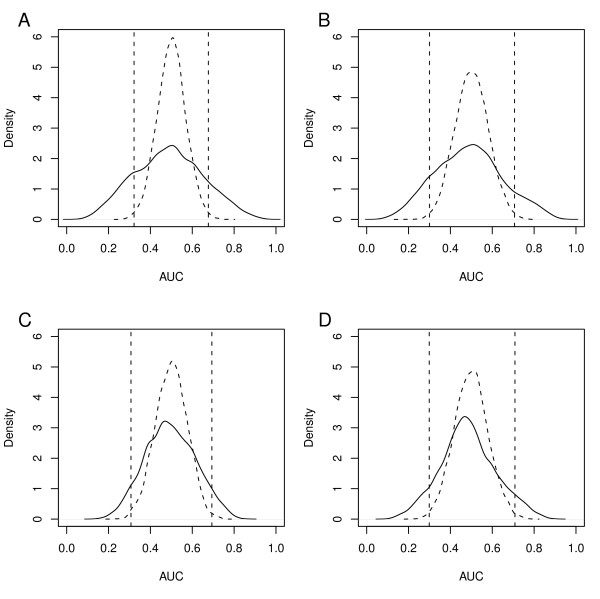
**Distribution of AUC values for all genes in a dataset**. AUC values in respect to MI-Status are plotted in solid lines for SanchezC data in A) and Stransky data in B). AUC values in respect to high grade (G3) are plotted in solid lines for SanchezC data in C) and the Stransky data in D). Distributions of AUC values for randomized versions are plotted in dashed lines and the intervals that contain 99% of randomized data are indicated with vertical dashed lines.

Two bladder cancer datasets, SanchezC and Stransky, were selected to explore the efficiency of predictor gene signatures based on ROC statistics. For each gene the association with muscle-invasiveness (MI) and tumor grade (G) as feature variables was estimated by calculating the AUC value in each dataset. The obtained AUC values ranged between 0.06 and 0.95, and 0.07 and 0.94 for MI, and between 0.14 and 0.85, and 0.10 and 0.89 for high grade (G3) in the SanchezC and Stransky data, respectively. The distribution of the AUC values deviated from the normal distribution and showed heavy tails (Figure 1). As a comparison, AUC values were also estimated in randomized versions of the SanchezC and Stransky datasets. In the randomized data 99% of the obtained AUC values were approximately between 0.7 and 0.3 for MI and tumor grade. A total of 31% and 23%, and 11% and 14% of genes fall outside these 99% borders for MI and Grade variables in the SanchezC and Stransky dataset, respectively. In contrast, 1% of genes are expected to fall outside for a randomized variable. The genes in excess to this 1% of false discovery genes are informative, and hence may be considered as the maximum size of a gene signature. Hence, a large proportion of genes show informative AUC values. Furthermore, as more genes are associated with MI than with grade, the major difference in bladder cancer phenotype seems to be associated with stage.

**Figure 2:**
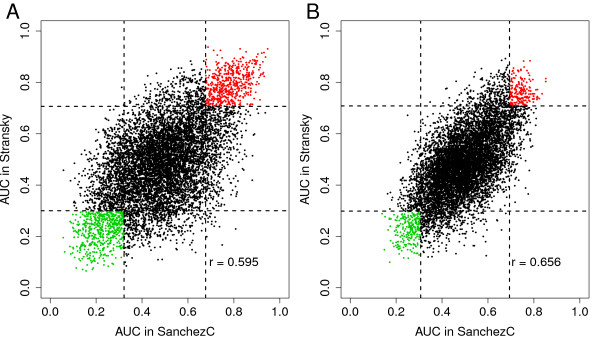
**Scatter plot of AUC values for 8518 genes shared by SanchezC and Stransky**. AUC values for MI-status are plotted in A and AUC values for grade are plotted in B. Genes with lower or higher AUC values than 99% of randomized data (dashed lines) in both datasets are depicted in green or red, respectively. r = Pearson correlation coefficient.

We then investigated the robustness of obtained AUC values by comparing AUC values from two different datasets. In Figure 2A we have plotted the AUC values for NMI/MI status and in Figure 2B the equivalent data for grade, for the SanchezC and Stransky data respectively. The correlations between the two datasets were moderate, 0.60 and 0.66, respectively. In fact, many genes change from significant AUC values (> 0.7 or <0.3) in one data set to insignificant values in the other, and even obtain AUC values in the opposite direction. Very few genes showed AUC values outside the 95% confidence interval of the randomized data in both datasets (Figure 2).

**Figure 3:**
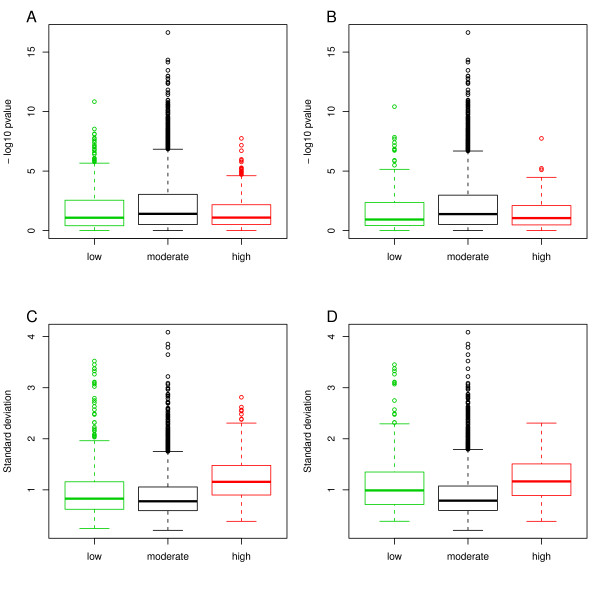
**Deviation from normal distribution and standard deviation of genes from SanchezC with significantly high or low AUC values in both datasets, SanchezC and Stransky**. A) Deviation from normal distribution of genes grouped by their association to MI in Figure 2A. B) Deviation from normal distribution of genes grouped by their association to grade in Figure 2B. C) Box plot for standard deviation of genes grouped by association to MI. D) Box plot for standard deviation of genes grouped by association to grade. -log10 p-value = logarithmic p-value of Shapiro test for normality.

We then investigated the possibility to identify robust predictor genes i.e., genes with informative AUC values in more than one dataset, by using more information during the feature selection process. We investigated deviations from a normal distribution as a possible additional criterion. In an optimal scenario an informative gene should show high expression in one and low expression in a second group in a two class situation and hence produce a bimodal distribution, or a distribution with a heavy tail. We used the Shapiro test for normality to identify genes with skewed or otherwise distorted distributions; the analysis revealed no differences between the two groups (Figure 3A and 3B). We investigated the variance of the informative genes as genes with large variance are expected to produce more robust results and did note a significant shift to larger variance for genes with AUC values >0.7. This shift was however too small to be of any practical use (Figure 3C and 3D). Similar results were obtained for the Stransky data (Additional file 3). From this we conclude that deviation from the normal distribution or large variance cannot be used to preselect genes with robust AUC values and hence no such functions were added to the software.

**Figure 4:**
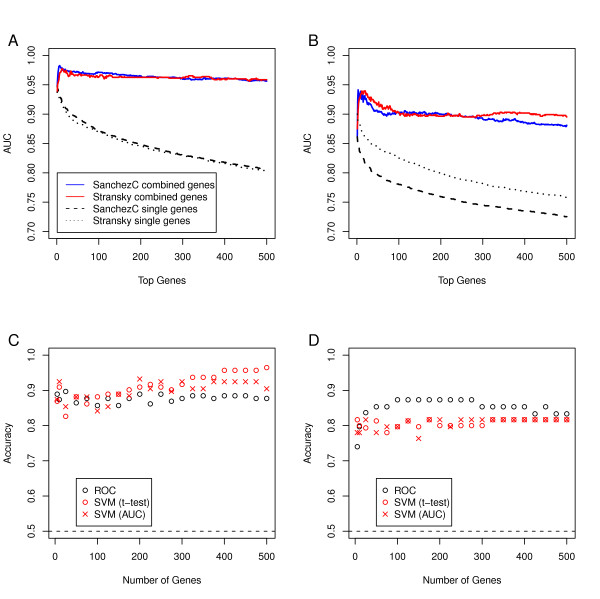
**Area under the Curve (AUC) values of the ranked single genes and of the metagene obtained from taking the mean expression of the single genes**. A) AUC for association to MI. B) AUC for association to grade. Classification performance of MI status for the metagene based predictor (ROC) and the SVM using LOOCV in the SanchezC (C) and Stransky data (D), respectively. For SVM we used two different features selection criteria, t-test and AUC. Accuracy = Balanced Accuracy (see Methods), dashed line = Balanced Accuracy obtained by random class assignment (= 0.5). ROC = metagene-based predictor. SVM = Support Vector Machine.

Gene expression is inherently noisy and random noise is expected to reduce the performance of predictors based on single genes. We therefore reasoned that the random noise effect could be counteracted by using the mean expression level for more than one gene. We consequently calculated the AUC values for all genes with respect to MI and grade in the Sanchez data. Before ranking, AUC values for genes with negative correlation, and hence showing AUC values <0.5, were inverted to values >0.5 by assigning AUC = 1-AUC^-^. We then designed gene signatures with increasing sizes by adding genes in order of their rank with steps of 1 at a time. For each signature the average expression level was calculated. In cases when genes showed negative association with MI or grade, the gene expression levels were inverted. The AUC values were then estimated using the expression levels of the created metagenes. Figure 4A and 4B show that the use of uni-variate gene expression average classifier results in considerably higher AUC values than those obtained for single and top ranking AUC genes. Furthermore, the AUC values turn stable when the metagenes become large enough.

### Classification performance

We next compared the prediction performance of AUC-based metagenes with the standard and frequently used SVM-method. To accomplish this we constructed AUC metagenes with increasing number of genes in steps of 5 up to 500 genes. For comparison, we applied the most differentially expressed genes as determined by a t-test or AUC values to SVM, also in increasing steps of 5 genes. The accuracies were estimated using LOOCV in both cases. In Figure 4C and 4D we tested the NMI/MI classification in the SanchezC and in the Stransky data, respectively. As can be seen the AUC metagene approach is as efficient as the SVM approach using both t-test and AUC as feature selection criteria; only slightly poorer in the SanchezC data, with accuracies close to 0.9 in both cases, but slightly better in the Stransky data, with accuracies close to 0.85 for the AUC metagene and 0.80 for the SVM approach. Hence, the simple AUC metagene approach seems to be as efficient as the more sophisticated SVM.

**Figure 5:**
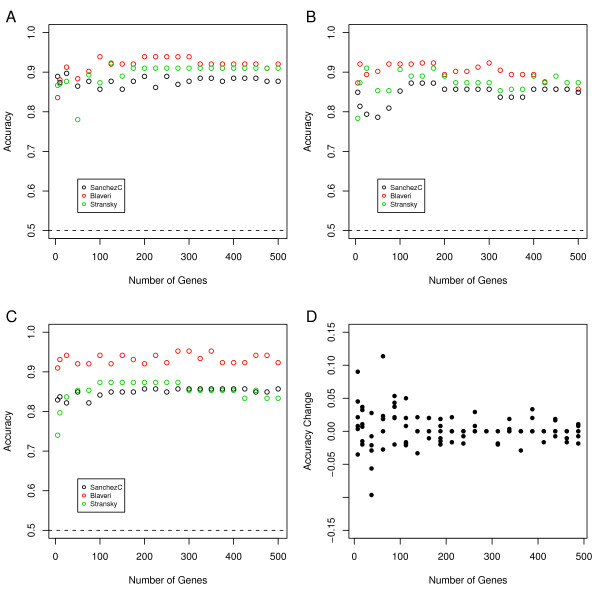
**Performance of the metagene-based classifier when applied to independent data**. A) Genes with highest AUC values of the SanchezC data are first tested by LOOCV in the SanchezC dataset (black circles) and then taken over to establish a classifier in the Stransky (green) and Blaveri (datasets), again by LOOCV. Gene signatures of 5-500 gene members are used. Balanced Accuracies are plotted; the dashed line at 0.5 indicates the balanced accuracy obtained by chance. B) and C): Procedure repeated with Blaveri and Stransky as training data, respectively. D) Changes in balanced accuracies from one gene signature size to the next biggest size are plotted for all balanced accuracies obtained in the validation datasets in A-C).

The robustness of the AUC metagene approach was then tested in independent data. For these purposes, we designed AUC metagene signatures with increasing sizes in one of the three datasets, SanchezC, Stransky, and Blaveri. The accuracy for each AUC metagene was first determined in the respective training set using LOOCV (Figure 5). Genes were then ranked according to AUC values using all cases in the training set and used as a source for producing gene signatures of increasing size applied to the independent data sets. These metagenes were then used to establish thresholds for maximum accuracies in the validation data sets within LOOCV loops. Hence, the signature genes used to produce the metagene were derived from the training set but the actual thresholds were derived from the validation sets. To estimate classification performance in the validation data sets we again performed LOOCV. In Figure 5A we have used metagenes obtained from the SanchezC dataset to predict NMI/MI in the Blaveri and Stransky datasets. As can be seen, when applied to the training set, i.e. the SanchezC dataset itself, accuracies close to 0.9 are obtained whereas in the Blaveri and Stransky accuracies slightly above 0.9 are obtained. Similar results were obtained for the other combinations of training and validation data, and hence, the AUC metagene approach is robust to independent data.

A close inspection of Figure 5A-C seems to indicate that signatures for MI in bladder cancer smaller than 100 genes are less robust i.e. show a large variance in accuracy when applied to validation data. To explore this further, we calculated the change in accuracies between each step of signature size for signatures derived from the same dataset. These values were then plotted (Figure 5D). It is obvious form Figure 5D that signatures smaller than 100 are sensitive to the composition of the signature, whereas signatures above 200 show robust performance, seen as small or no changes in accuracies for different AUC metagenes. From this one may conclude that gene signatures of at least 150 genes are needed to produce robust predictors of NMI/MI in bladder.

The metagene classifier approach was then evaluated further using two approaches. First, we evaluated the algorithm performance regarding different endpoints. For this purpose we used three breast cancer datasets (vandeVijver, WangY, Sotiriou), three neuroblastoma datasets (WangQ, JanoueixL, Attiyeh) and two lung cancer datasets (Angulo, Takeuchi), and six different endpoints; ER status, tumor grade, tumor size, tumor stage, MYCN amplification status, and histological subtype. Second, we compared the uni-variate gene expression average classifier with 12 alternative classification algorithms using two feature selection procedures.

In these comparisons we predetermined the number of genes to be included in the metagene to 50, as this is close to the median number of genes included in published gene signatures. For each endpoint the selected features (genes) were determined in one dataset and then applied to the remaining ones (Table 1). For classification of e.g. ER status in breast cancer the gene list was derived from one dataset and then applied to the two remaining ones. This procedure was repeated using each dataset for feature selection resulting in a total of six tests. Overall the accuracies were high, ranging from 0.77 to 0.94 and similar to those obtained by SVM (range 0.71 to 0.95) when applied to validation data. Instances resulting in lower range accuracies using the metagene predictor where also low when using the SVM approach, indicating that the obtained lower accuracies were dependent on dataset and not on the algorithm used. Tumor grade was only available for two breast cancer datasets, vandeVijver and Sotiriou. Grade was predicted in validation data with accuracies ranging from 0.82 to 0.85 using the metagene predictor, similar to what was obtained by SVM (Table 1). The uni-variate gene exression average classifier could also faithfully predict MYCN status in neuroblastoma and histopathological subtype in lung cancers. Tumor stage in neuroblastoma and tumor grade in lung cancers were predicted with lower but significant accuracies. As can be seen from the computed average accuracies for each phenotype, the simple metagene predictor on average performs just as well, or better, than the more complex SVM approach. All predictor evaluations were repeated using 10 and 200 member metagenes, the obtained accuracies were however not influenced by the size of the metagenes (Additional file 4).

**Table 1:** Balanced accuracies of prediction obtained in various phenotypes using the metagene classifier (ROC) and linear SVM (SVM).

Phenotype/ Method	Training^1^	Validation	Validation	Training	Validation	Validation	Training	Validation	Validation	Mean
**ER^2 ^(BC)**	vandeVijver	WangY	Sotiriou	WangY	vandeVijver	Sotiriou	Sotiriou	vandeVijver	WangY	
ROC	*0.95*	**0.89**	**0.74**	*0.89*	**0.94**	**0.77**	*0.77*	**0.90**	**0.88**	0.85
SVM	*0.94*	**0.82**	**0.71**	*0.82*	**0.95**	**0.73**	*0.69*	**0.95**	**0.82**	0.83
**Grade^3 ^(BC)**	vandeVijver	Sotiriou		Sotiriou	vandeVijver					
ROC	*0.79*	**0.85**		*0.84*	**0.82**					0.84
SVM	*0.72*	**0.78**		*0.84*	**0.78**					0.78
**Size^4 ^(BC)**	vandeVijver	Sotiriou		Sotiriou	vandeVijver					
ROC	*0.63*	**0.65**		*0.70*	**0.60**					0.63
SVM	*0.57*	**0.51**		*0.61*	**0.48**					0.49
**MYCN^5^(NB)**	WangQ	JanoueixL	Attiyeh	JanoueixL	WangQ	Attiyeh	Attiyeh	WangQ	JanoueixL	
ROC	*0.94*	**0.91**	**0.82**	*0.87*	**0.89**	**0.79**	*0.81*	**0.95**	**0.89**	0.88
SVM	*0.98*	**0.83**	**0.75**	*0.88*	**0.97**	**0.67**	*0.85*	**0.98**	**0.87**	0.84
**Stage^6 ^(NB)**	WangQ	JanoueixL	Attiyeh	JanoueixL	WangQ	Attiyeh	Attiyeh	WangQ	JanoueixL	
ROC	*0.74*	**0.58**	**0.82**	*0.66*	**0.65**	**0.70**	*0.69*	**0.62**	**0.48**	0.64
SVM	*0.79*	**0.58**	**0.74**	*0.61*	**0.73**	**0.61**	*0.60*	**0.72**	**0.54**	0.65
**AD/SQ^7^(LC)**	Angulo	Takeuchi		Takeuchi	Angulo					
ROC	*0.93*	**0.96**		*0.95*	**0.96**					0.96
SVM	*0.89*	**0.91**		*0.93*	**0.84**					0.88
**Grade^8^(LC)**	Angulo	Takeuchi		Takeuchi	Angulo					
ROC	*0.67*	**0.69**		*0.69*	**0.68**					0.68
SVM	*0.63*	**0.64**		*0.63*	**0.59**					0.61

^1 ^Accuracies given in italics indicates accuracies obtained in the training set using LOOCV

^2 ^ER+ vs. ER-

^3 ^grade 1 vs. grade 3

^4 ^tumors < = 2 cm vs. tumors > 2 cm

^5 ^MYCN amplification positive vs. MYCN amplification negative

^6 ^stage 1 and 2 vs. stage 3 and 4

^7 ^adenocarcinoma vs. squamous cell carcinoma

^8 ^grade 1 vs. grade 2 and 3

Abbreviations; BC = Breast Cancer, NB = Neuroblastoma, LC = Lung Cancer

For the extensive comparison with other classification algorithms we limited the analysis to ER status in breast cancer, MYCN status in neuroblastoma, and adenocarcinoma/squamous cell carcinoma status in lung cancer using a t-test as feature selection criteria (Table 2). For comparison we also used AUC as feature selection criteria (Additional file 5). As can be seen in Table 2, all classification algorithms show similar performance. The performance is more dependent on the endpoint/dataset than the actual algorithms used. Furthermore, simple classifiers such as the discriminant analysis methods (lda, dlda, slda) perform just as well as the more sophisticated ones, such as the neural network algorithm neuralnet. Our uni-variate gene expression average classifier shows good performance, ranking first, third, and fifth in the lung cancer, breast cancer and neuroblastoma datasets, respectively. Almost identical results were obtained when using AUC as feature selection criteria (Additional file 5).

**Table 2:** Balanced accuracies of prediction using the metagene classifier (ROC) and various classification algorithms and a t-test as feature selection criteria.

Phenotype/ Method	Training^1^	Validation	Validation	Training	Validation	Validation	Training	Validation	Validation	Mean
**ER^2 ^(BC)**	vandeVijver	WangY	Sotiriou	WangY	vandeVijver	Sotiriou	Sotiriou	vandeVijver	WangY	
ROC^5^	*0.95*	**0.89**	**0.74**	*0.89*	**0.94**	**0.77**	*0.77*	**0.90**	**0.88**	0.85
SVM	*0.94*	**0.82**	**0.71**	*0.82*	**0.95**	**0.73**	*0.69*	**0.95**	**0.82**	0.83
SVMradial	*0.94*	**0.86**	**0.70**	*0.87*	**0.93**	**0.73**	*0.76*	**0.93**	**0.84**	0.83
SVMpoly	*0.92*	**0.80**	**0.67**	*0.77*	**0.89**	**0.65**	*0.75*	**0.84**	**0.72**	0.76
knn	*0.94*	**0.83**	**0.70**	*0.84*	**0.93**	**0.78**	*0.77*	**0.95**	**0.78**	0.83
rforest	*0.98*	**0.85**	**0.73**	*0.84*	**0.93**	**0.73**	*0.76*	**0.98**	**0.83**	0.84
rpart	*1.00*	**0.79**	**0.61**	*0.82*	**0.90**	**0.63**	*0.72*	**1.00**	**0.75**	0.78
bagging	*1.00*	**0.84**	**0.72**	*0.86*	**0.91**	**0.72**	*0.81*	**1.00**	**0.84**	0.84
lda	*0.95*	**0.85**	**0.76**	*0.86*	**0.94**	**0.73**	*0.76*	**0.96**	**0.83**	0.84
dlda	*0.96*	**0.89**	**0.79**	*0.88*	**0.94**	**0.77**	*0.87*	**0.94**	**0.85**	0.86
slda	*0.94*	**0.86**	**0.74**	*0.85*	**0.94**	**0.77**	*0.77*	**0.94**	**0.85**	0.85
neuralnet	*0.94*	**0.78**	**0.69**	*0.81*	**0.92**	**0.72**	*0.76*	**0.97**	**0.80**	0.81
ncc	*0.96*	**0.89**	**0.78**	*0.88*	**0.94**	**0.75**	*0.82*	**0.95**	**0.87**	0.86
**MYCN^3 ^(NB)**	WangQ	JanoueixL	Attiyeh	JanoueixL	WangQ	Attiyeh	Attiyeh	WangQ	JanoueixL	
ROC	*0.94*	**0.91**	**0.82**	*0.87*	**0.89**	**0.79**	*0.81*	**0.95**	**0.89**	0.88
SVM	*0.98*	**0.83**	**0.75**	*0.88*	**0.97**	**0.67**	*0.85*	**0.98**	**0.87**	0.84
SVMradial	*0.95*	**0.93**	**0.82**	*0.93*	**0.94**	**0.81**	*0.87*	**0.95**	**0.86**	0.88
SVMpoly	*0.95*	**0.86**	**0.74**	*0.79*	**0.88**	**0.69**	*0.82*	**0.90**	**0.79**	0.81
knn	*0.95*	**0.93**	**0.86**	*0.88*	**0.92**	**0.85**	*0.86*	**0.98**	**0.85**	0.90
rforest	*0.95*	**0.93**	**0.80**	*0.91*	**0.94**	**0.76**	*0.84*	**0.95**	**0.88**	0.88
rpart	*1.00*	**0.93**	**0.90**	*0.83*	**0.89**	**0.72**	*0.90*	**1.00**	**0.72**	0.86
bagging	*1.00*	**0.90**	**0.89**	*0.81*	**0.91**	**0.72**	*0.87*	**1.00**	**0.86**	0.88
lda	*1.00*	**0.70**	**0.83**	*0.69*	**0.94**	**0.73**	*0.86*	**0.98**	**0.65**	0.80
dlda	*0.94*	**0.90**	**0.86**	*0.90*	**0.94**	**0.86**	*0.84*	**0.95**	**0.85**	0.89
slda	*0.95*	**0.93**	**0.85**	*0.93*	**0.98**	**0.83**	*0.89*	**0.98**	**0.91**	0.91
neuralnet	*0.99*	**0.81**	**0.89**	*0.85*	**0.96**	**0.80**	*0.88*	**0.97**	**0.82**	0.87
ncc	*0.94*	**0.91**	**0.86**	*0.93*	**0.94**	**0.85**	*0.86*	**0.98**	**0.87**	0.90
**AD/SQ^4 ^(LC)**	Angulo	Takeuchi		Takeuchi	Angulo					
ROC	*0.93*	**0.96**		*0.95*	**0.96**					0.96
SVM	*0.89*	**0.91**		*0.93*	**0.84**					0.88
SVMradial	*0.97*	**0.93**		*0.96*	**0.96**					0.95
SVMpoly	*0.89*	**0.92**		*0.90*	**0.96**					0.94
knn	*0.94*	**0.94**		*0.95*	**0.94**					0.94
rforest	*0.94*	**0.95**		*0.96*	**0.94**					0.94
rpart	*0.86*	**0.93**		*0.95*	**0.82**					0.87
bagging	*0.91*	**0.96**		*0.95*	**0.93**					0.94
lda	*0.80*	**0.91**		*0.93*	**0.83**					0.87
dlda	*0.94*	**0.95**		*0.95*	**0.92**					0.93
slda	*0.93*	**0.95**		*0.95*	**0.96**					0.96
neuralnet	*0.91*	**0.95**		*0.94*	**0.92**					0.94
ncc	*0.93*	**0.96**		*0.95*	**0.93**					0.95

^1 ^Accuracies given in italics indicates accuracies obtained in the training set using LOOCV

^2 ^ER+ vs. ER-

^3 ^MYCN amplification positive vs. MYCN amplification negative

^4 ^adenocarcinoma vs. squamous cell carcinoma

^5 ^the metagene classifier uses AUC values as feature selection criteria.

Abbreviations; BC = Breast Cancer, NB = Neuroblastoma, LC = Lung Cancer

## Conclusions

We have developed a new algorithm for tumor classification based on the formation of gene expression metagenes and of feature selection using AUC values obtained by ROC analysis. This simple classification algorithm shows good performance and is robust in independent validation data. The described approach has the potential to be a valuable complement to algorithms based on alternative principles.

## Availability and Requirements

• **Project name: ***rocc*

• **Project homepage: **http://cran.r-project.org/web/packages/rocc/index.html

• **Operating systems: **Platform independent

• **Programming language: **R

• **Other requirements: **R 2.9.2 or higher is recommended. The installation of the R package *ROCR *is required.

• **License: **GPL2 or higher

• **Any restrictions to use by non-academics: **none

## Competing interests

The authors declare that they have no competing interests.

## Authors' contributions

ML performed the majority of the computations, wrote the R package and parts of the manuscript. AF and TR performed the first evaluations of the predictor algorithm. MH conceived and planned the investigation, and wrote parts of the manuscript. All authors read and approved the final manuscript.

## Pre-publication history

The pre-publication history for this paper can be accessed here:

http://www.biomedcentral.com/1471-2407/10/532/prepub

## Supplementary Material

Additional file 1 **Description of the used cancer datasets**. Click here for file

Additional file 2 **Scheme of the generation of metagenes**. Click here for file

Additional file 3 **Deviation from normal distribution and standard deviation of genes from Stransky with significantly high or low AUC values in both datasets, SanchezC and Stransky**. A) Deviation from normal distribution of genes grouped by their association to MI in Figure 2A. B) Deviation from normal distribution of genes grouped by their association to grade in Figure 2B. C) Box plot for standard deviation of genes grouped by association to MI. D) Box plot for standard deviation of genes grouped by association to grade. -log10 p-value = logarithmic p-value of Shapiro test for normality. Click here for file

Additional file 4 **Balanced accuracies of prediction obtained using the metagene-based classifier and Support Vector Machine for gene signatures that consist of 10, 50 and 200 genes**. Click here for file

Additional file 5 **Balanced accuracies of prediction using the metagene classifier (ROC) and various classification algorithms and AUC as feature selection criteria**. Click here for file

## References

[B1] Khan J Wei JS Ringner M Saal LH Ladanyi M Westermann F Berthold F Schwab M Antonescu CR Peterson C Classification and diagnostic prediction of cancers using gene expression profiling and artificial neural networks Nat Med 2001 7 673 679 10.1038/89044 11385503 PMC1282521

[B2] Theilhaber J Connolly T Roman-Roman S Bushnell S Jackson A Call K Garcia T Baron R Finding genes in the C2C12 osteogenic pathway by k-nearest-neighbor classification of expression data Genome Res 2002 12 165 176 10.1101/gr.182601 11779842 PMC155256

[B3] Tamayo P Slonim D Mesirov J Zhu Q Kitareewan S Dmitrovsky E Lander ES Golub TR Interpreting patterns of gene expression with self-organizing maps: methods and application to hematopoietic differentiation Proc Natl Acad Sci USA 1999 96 2907 2912 10.1073/pnas.96.6.2907 10077610 PMC15868

[B4] Dudoit S Fridlyand J Speed TP Comparison of Discrimination Methods for the Classification of Tumors Using Gene Expression Data J Am Stat Assoc 2002 97 77 87 10.1198/016214502753479248

[B5] Pepe MS Longton G Anderson GL Schummer M Selecting differentially expressed genes from microarray experiments Biometrics 2003 59 133 142 10.1111/1541-0420.00016 12762450

[B6] Jaeger J Sengupta R Ruzzo WL Improved gene selection for classification of microarrays Pac Symp Biocomput 2003 53 64 12603017 10.1142/9789812776303_0006

[B7] Brown MP Grundy WN Lin D Cristianini N Sugnet CW Furey TS Ares M Jr Haussler D Knowledge-based analysis of microarray gene expression data by using support vector machines Proc Natl Acad Sci USA 2000 97 262 267 10.1073/pnas.97.1.262 10618406 PMC26651

[B8] Tibshirani R Hastie T Narasimhan B Chu G Diagnosis of multiple cancer types by shrunken centroids of gene expression Proc Natl Acad Sci USA 2002 99 6567 6572 10.1073/pnas.082099299 12011421 PMC124443

[B9] Shi L Campbell G Jones WD Campagne F Wen Z Walker SJ Su Z Chu TM Goodsaid FM Pusztai L The MicroArray Quality Control (MAQC)-II study of common practices for the development and validation of microarray-based predictive models Nat Biotechnol 2010 28 827 838 10.1038/nbt.1665 20676074 PMC3315840

[B10] Sanchez-Carbayo M Socci ND Lozano J Saint F Cordon-Cardo C Defining molecular profiles of poor outcome in patients with invasive bladder cancer using oligonucleotide microarrays J Clin Oncol 2006 24 778 789 10.1200/JCO.2005.03.2375 16432078

[B11] Stransky N Vallot C Reyal F Bernard-Pierrot I de Medina SG Segraves R de RY Elvin P Cassidy A Spraggon C Regional copy number-independent deregulation of transcription in cancer Nat Genet 2006 38 1386 1396 10.1038/ng1923 17099711

[B12] Blaveri E Simko JP Korkola JE Brewer JL Baehner F Mehta K Devries S Koppie T Pejavar S Carroll P Bladder cancer outcome and subtype classification by gene expression Clin Cancer Res 2005 11 4044 4055 10.1158/1078-0432.CCR-04-2409 15930339

[B13] Edgar R Domrachev M Lash AE Gene Expression Omnibus: NCBI gene expression and hybridization array data repository Nucleic Acids Res 2002 30 207 210 10.1093/nar/30.1.207 11752295 PMC99122

[B14] van de Vijver MJ He YD van't Veer LJ Dai H Hart AA Voskuil DW Schreiber GJ Peterse JL Roberts C Marton MJ A gene-expression signature as a predictor of survival in breast cancer N Engl J Med 2002 347 1999 2009 10.1056/NEJMoa021967 12490681

[B15] Smyth GK Linear models and empirical bayes methods for assessing differential expression in microarray experiments Stat Appl Genet Mol Biol 2004 3 Article3 16646809 10.2202/1544-6115.1027

[B16] Gautier L Cope L Bolstad BM Irizarry RA affy--analysis of Affymetrix GeneChip data at the probe level Bioinformatics 2004 20 307 315 10.1093/bioinformatics/btg405 14960456

[B17] Troyanskaya O Cantor M Sherlock G Brown P Hastie T Tibshirani R Botstein D Altman RB Missing value estimation methods for DNA microarrays Bioinformatics 2001 17 520 525 10.1093/bioinformatics/17.6.520 11395428

[B18] Eyre TA Ducluzeau F Sneddon TP Povey S Bruford EA Lush MJ The HUGO Gene Nomenclature Database, 2006 updates Nucleic Acids Res 2006 34 D319 D321 10.1093/nar/gkj147 16381876 PMC1347509

[B19] Sing T Sander O Beerenwinkel N Lengauer T ROCR: visualizing classifier performance in R Bioinformatics 2005 21 3940 3941 10.1093/bioinformatics/bti623 16096348

[B20] Rosenwald A Wright G Chan WC Connors JM Campo E Fisher RI Gascoyne RD Muller-Hermelink HK Smeland EB Giltnane JM The use of molecular profiling to predict survival after chemotherapy for diffuse large-B-cell lymphoma N Engl J Med 2002 346 1937 1947 10.1056/NEJMoa012914 12075054

[B21] Statnikov A Aliferis CF Tsamardinos I Hardin D Levy S A comprehensive evaluation of multicategory classification methods for microarray gene expression cancer diagnosis Bioinformatics 2005 21 631 643 10.1093/bioinformatics/bti033 15374862

[B22] Simon R Lam A Li MC Ngan M Menenzes S Zhao Y Analysis of Gene Expression Data Using BRB-Array Tools Cancer Inform 2007 3 11 17 19455231 PMC2675854

[B23] Noble WS What is a support vector machine? Nat Biotechnol 2006 24 1565 1567 10.1038/nbt1206-1565 17160063

[B24] R package e1071 http://cran.r-project.org/web/packages/e1071/index.html

[B25] R package MLInterfaces http://www.bioconductor.org/packages/release/bioc/html/MLInterfaces.html

[B26] Velez DR White BC Motsinger AA Bush WS Ritchie MD Williams SM Moore JH A balanced accuracy function for epistasis modeling in imbalanced datasets using multifactor dimensionality reduction Genet Epidemiol 2007 31 306 315 10.1002/gepi.20211 17323372

