# Supplementary figures and images for: Robust assignment of cancer subtypes from expression data using a uni-variate gene expression average as classifier
Source: BMC Cancer. 2010 Oct 6;10:532. doi: 10.1186/1471-2407-10-532 (PMC2966465; doi:10.1186/1471-2407-10-532)

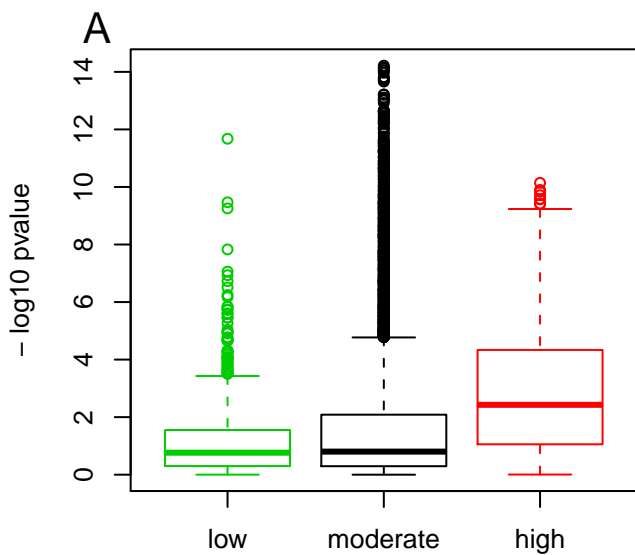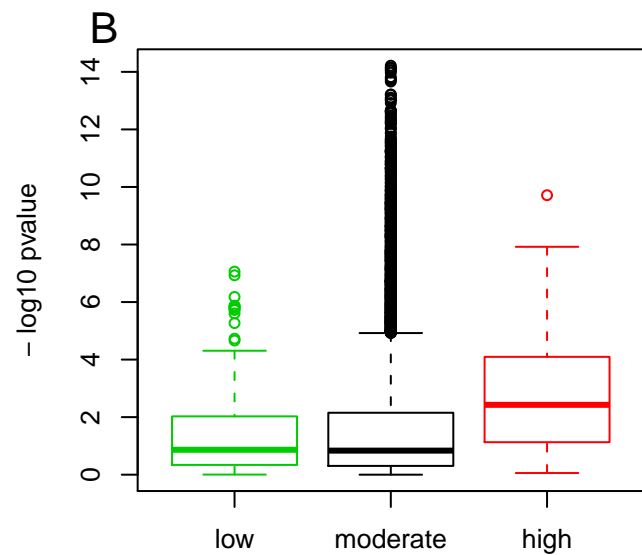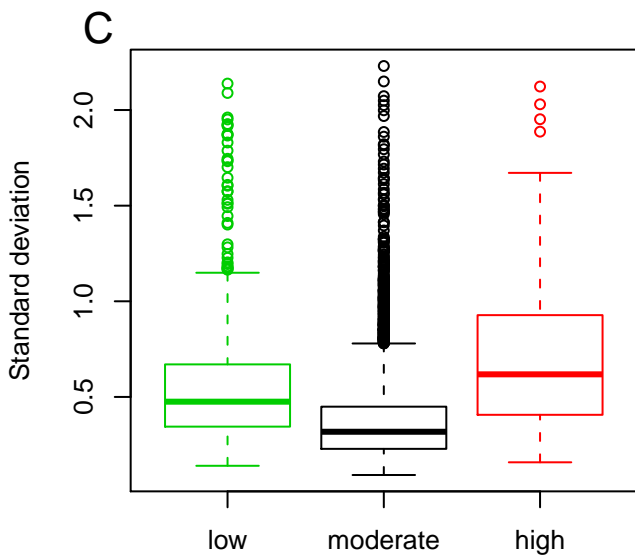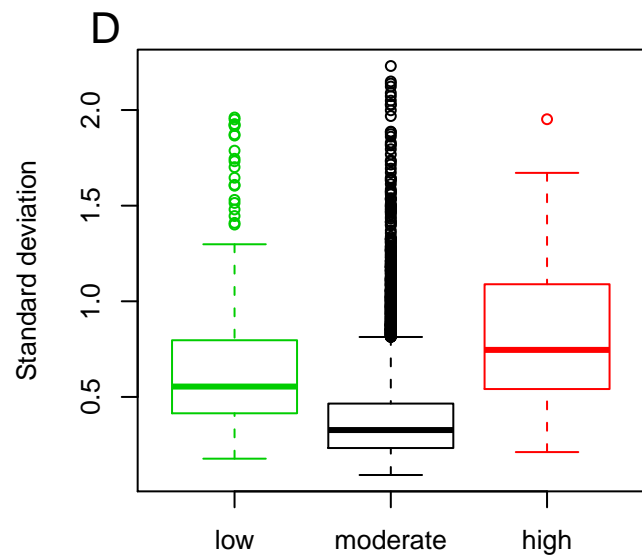

Supplement: Additional file 3 — Deviation from normal distribution and standard deviation of genes from Stransky with significantly high or low AUC values in both datasets, SanchezC and Stransky. A) Deviation from normal distribution of genes grouped by their association to MI in Figure 2A. B) Deviation from normal distribution of genes grouped by their association to grade in Figure 2B. C) Box plot for standard deviation of genes grouped by association to MI. D) Box plot for standard deviation of genes grouped by association to grade. -log10 p-value = logarithmic p-value of Shapiro test for normality. [file 1471-2407-10-532-S3.PDF]
